# Ptaquiloside in Irish Bracken Ferns and Receiving Waters, with Implications for Land Managers

**DOI:** 10.3390/molecules21050543

**Published:** 2016-04-26

**Authors:** Connie O’Driscoll, Carmel Ramwell, Brendan Harhen, Liam Morrison, Frederik Clauson-Kaas, Hans Christian B. Hansen, Graeme Campbell, Jerome Sheahan, Bruce Misstear, Liwen Xiao

**Affiliations:** 1 Department of Civil, Structural and Environmental Engineering, Trinity College Dublin, Dublin 2, Ireland; BMISSTER@tcd.ie; 2 Department of Civil Engineering, National University of Ireland, Galway H91 HX31, Ireland; 3 Centre for Chemical Safety and Stewardship, Fera Science Ltd., Sand Hutton, York YO41 1LZ, UK; Carmel.Ramwell@fera.co.uk; 4 National Centre for Biomedical Engineering Science, National University of Ireland, Galway H91 HX31, Ireland; brendan.harhen@nuigalway.ie; 5 Earth and Ocean Sciences, School of Natural Sciences and Ryan Institute, National University of Ireland, Galway H91 HX31, Ireland; liam.morrison@nuigalway.ie; 6 Department of Plant and Environmental Sciences, University of Copenhagen, Frederiksberg DK-1871, Denmark; pfck@plen.ku.dk (F.C.-K.); haha@plen.ku.dk (H.C.B.H.); 7 College of Agriculture Food and Rural Enterprise Greenmount Campus, 45 Tirgracy Road, Muckamore, Antrim BT41 4PS, UK; Graeme.Campbell@dardni.gov.uk; 8 School of Mathematics, Statistics and Applied Mathematics, National University of Ireland, Galway H91 HX31, Ireland; jerome.sheahan@nuigalway.ie

**Keywords:** ptaquiloside, bracken, drinking water, phytochemicals, land management, Ireland

## Abstract

Ptaquiloside, along with other natural phytotoxins, is receiving increased attention from scientists and land use managers. There is an urgent need to increase empirical evidence to understand the scale of phytotoxin mobilisation and potential to enter into the environment. In this study the risk of ptaquiloside to drinking water was assessed by quantifying ptaquiloside in the receiving waters at three drinking water abstraction sites across Ireland and in bracken fronds surrounding the abstraction sites. We also investigated the impact of different management regimes (spraying, cutting and rolling) on ptaquiloside concentrations at plot-scale in six locations in Northern Ireland, UK. Ptaquiloside concentrations were determined using recent advances in the use of LC-MS for the detection and quantification of ptaquiloside. The results indicate that ptaquiloside is present in bracken stands surrounding drinking water abstractions in Ireland, and ptaquiloside concentrations were also observed in the receiving waters. Furthermore, spraying was found to be the most effective bracken management regime observed in terms of reducing ptaquiloside load. Increased awareness is vital on the implications of managing land with extensive bracken stands.

## 1. Introduction

**Figure 1:**
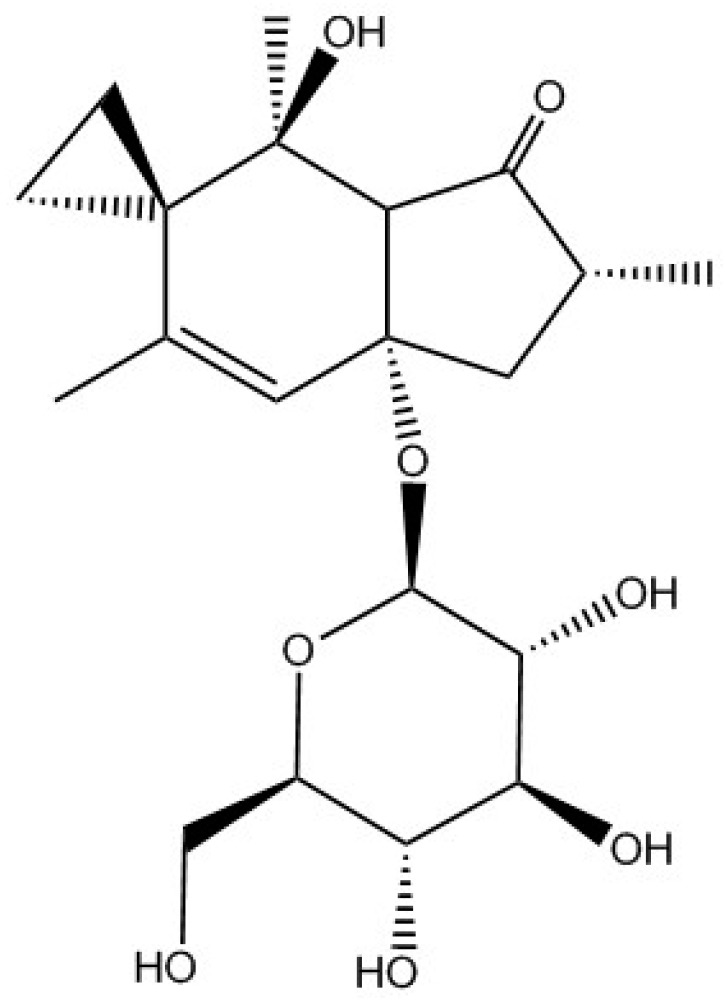
Chemical structure of ptaquiloside.

Phytochemical armament is an effective and widespread defense strategy in promoting direct vegetative survival, and it is a relatively new area of scientific interest mostly only becoming known about in the past 40 years [1]. Bracken fern (*Pteridium* spp.) has a vast array of diverse phytochemical armaments, containing a substantial number of potentially harmful substances including illudane, ptaquiloside and protoilludane glycosides [1]. Ptaquiloside (Figure 1), a highly water soluble phytotoxin, was first isolated in 1983 and identified as having carcinogenic, mutagenic and clastogenic behaviours [2,3]. More recently ptesculentoside and caudatoside have been isolated in quantities comparable to ptaquiloside and they have been identified as having similar chemical reactivity and presumably biological activity [4]. These compounds are unstable in mildly acidic and alkaline media [5] and are heat sensitive [6,7,8]. They hydrolyse to form non-toxic hydrophobic pterosins [9,10,11]. Further advances in metabolite detection using mass spectrometry will enable quantification of ptaquiloside and pterosin B at environmentally relevant concentrations [12].

Bracken fern (*Pteridium* spp.) has a cosmopolitan distribution and is the fifth most abundant weed species in the world [13]. Historically, bracken was utilised as compost, thatch, animal bedding and a source of potash [14]. Presently, the value of bracken is largely restricted to its ecological role (its use in palaeo- and neo-botany [1] and providing habitats for several priority species [14]), with only a small revival in its use as compost in the UK [15]; for the most part, however, bracken is considered a problematic weed species. Bracken has an extremely high rate of reproduction and re-growth owing to its extensive rhizomes, and can rapidly adapt to ecological conditions taking advantage of abandoned, newly cut or burned areas [16]. The percentage of land area covered by bracken is reported to be trending upwards with an average annual increase estimated at one percent in the UK [16]. This invasion is set to become more aggressive considering that a ban on *N*-(4-aminophenyl) sulfonylcarbamic acid methyl ester (asulam), the most effective herbicide for bracken control, came into effect in 2012 in the EU. Internationally, the UK is the only region to have completed national scale assessments of bracken coverage as part of their Countryside Survey, the latest being in 2007 [17], and so currently the international scale of bracken invasion cannot be accurately quantified.

Ptaquiloside can contaminate food supplies via several potential direct or indirect pathways [16]. Diseases encountered in sheep and cattle grazing in bracken areas include thiamine deficiency syndrome, acute bracken poisoning, progressive retinal degeneration (sheep only), and tumours in the urinary bladder and upper gastrointestinal tract [16]. Consumption of young croziers, inhalation of bracken spores, ptaquiloside-contaminated milk, ptaquiloside residues in meat and ptaquiloside contamination of drinking water have been proposed and investigated as human pathway exposures [16]. The infrequency of sporulation coupled with the low content of ptaquiloside in bracken spores means that exposure of ptaquiloside through airborne bracken spores is considered insignificant [18]. Recent research indicates that drinking water abstraction in bracken infested areas is a potential concern [19]; however, there is a lack of empirical evidence to support this. In addition to toxicological effects, invasion by bracken reduces the amount and quality of land available for grazing, conservation and recreation [20]. Common management practices include spraying, rolling, cutting, livestock grazing or burning, and combinations of these strategies. Limited studies have investigated whether different bracken control measures potentially impact on ptaquiloside concentrations in the plant. Fronds of *Pteridium* *esculentum* subjected to mowing or grazing contained higher levels of ptaquiloside compared to non-disturbed fronds [21], and similarly, higher ptaquiloside concentrations occurred in fronds after cutting when compared to uncut fronds [22]. It has been suggested that an increase in ptaquiloside concentration would not necessarily equate to increased ptaquiloside loading, as weakened plants would likely produce a lower biomass [23]. Additionally, higher concentrations of ptaquiloside in bracken that has received management interventions could be due to the earlier growth stage of fronds [22].

In north-western Europe, bracken fronds typically surface from underground roots in mid-May reaching maturity from late-July to early-September [22]. Total biomass can vary between countries from 460 g·m^−2^–1000 g·m^−2^ (Denmark [24] and the UK [25] respectively). Total biomass variation can also arise between sites with deep soils (940 g·m^−2^) *versus* those with shallow soils (430 g·m^−2^ [26]) and altitude (a 2-fold decrease in biomass from 346–228 m.a.s.l. [27]). Similarly, ptaquiloside accumulation in bracken is extremely variable and subject to chemotype, location in the plant and growth stage [28]. The ptaquiloside concentration in bracken fronds is reported to be highest in spring in newly surfaced croziers and can range from 0–9800 µg·g^−1^ [29]. Rhizome concentrations range from 2–7000 µg·g^−1^ [30] with concentrations typically peaking after fronds have reached maturity. Ptaquiloside concentration in bracken spores ranges from 4.5–23.5 µg·g^−1^ [18]. Bracken litter concentrations of ptaquiloside are reported to range from 0.09–23.5 µg·g^−1^ [28].

In Ireland, bracken is included in the Habitat Survey Guidelines [31]; however there is limited bracken percentage coverage data for Ireland [32] and historical trends have not been researched or quantified. There is therefore a need to quantify the national risk posed by bracken toxins to drinking water supplies. Furthermore, with an EU ban on asulam, there is limited knowledge on the effects of different bracken management options on ptaquiloside concentrations. The objectives of the present study were to contribute to this knowledge gap by 1) assessing the potential risk of ptaquiloside to drinking water by quantifying ptaquiloside in the receiving water at three drinking water abstraction sites across the Ireland and in bracken vegetation surrounding the abstraction site, and 2) investigating ptaquiloside concentrations in bracken stands with different management regimes at plot-scale in six locations in Northern Ireland, UK.

## 2. Results and Discussion

### 2.1. Study Site Characterisation

**Figure 2:**
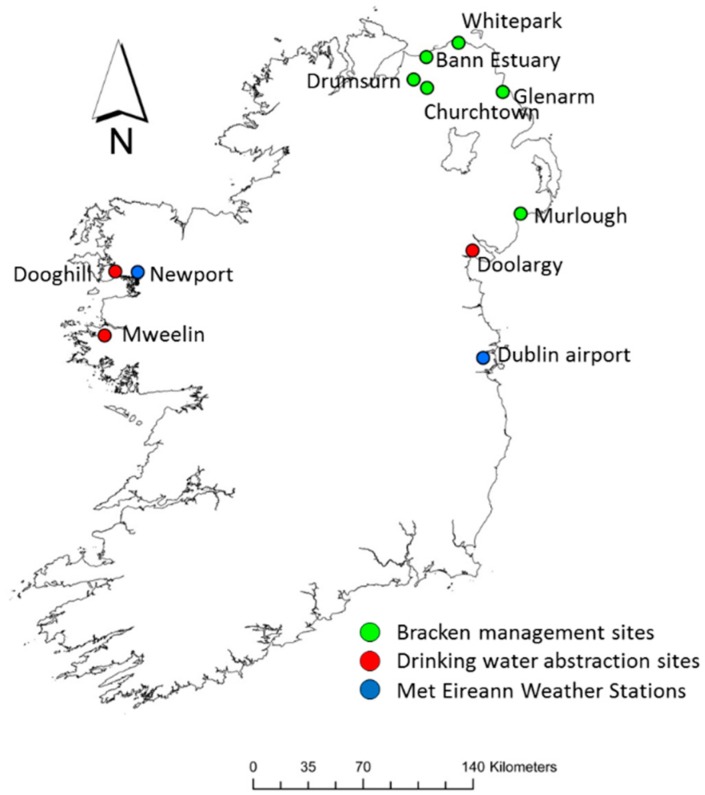
Map of the island of Ireland with locations of study sites and weather stations.

This study was based on nine bracken stands, six in Northern Ireland, UK, two on the west coast of Ireland and one on the east coast (Figure 2; Table 1). The stands were situated at a range of altitudes (6–148 m.a.s.l.).

The main land use at these locations was cattle and sheep grazing and the catchments receive an average annual precipitation of 900–2000 mm·yr^−1^ (www.met.ie). All bracken stands in the study comprised *Pteridium aquilinum* (L.) Kuhn. Of the three water abstraction sites, one was a surface water stream (Dooghill) and two were private spring wells (Doolargy and Mweelin). All water sources were in close proximity to the bracken stands (<50 m). The underlying soils around the surface water stream (Dooghill) were described as “peaty gleys” (http://gis.epa.ie/Envision) which are considered as poor percolation soils and more susceptible to risk from surface runoff contaminants. Similarly, both spring wells had “peaty gleys” surrounding them and were considered to have “extreme” groundwater vulnerability (www.gsi.ie). Groundwater vulnerability is assessed on: 1) the permeability of the subsoils overlying the groundwater; 2) the thickness of the unsaturated zone through which the contaminant moves; and 3) the type of recharge - either diffuse (direct recharge) or point (indirect recharge, such as via swallow holes in karst areas). Soil pH was slightly acidic (5.30–7.5) and nutrient poor with phosphorus (P) ranging from <1–3 mg·kg^−1^ of dry weight, potassium (K) ranging from 35–96 mg·kg^−1^ of dry weight and magnesium (Mg) ranging from 78–176 mg·kg^−1^ of dry weight (Table 1).

### 2.2. Ptaquiloside in Bracken Fronds Collected from the Drinking Water Abstraction Sites

Above-ground frond growth began in late-May to early-June. Ptaquiloside was detected in all frond samples analysed from June to November 2015 (Table 2). The highest ptaquiloside concentration was observed at Doolargy in June 2015 (297 µg·g^−1^).

**Figure 4:**
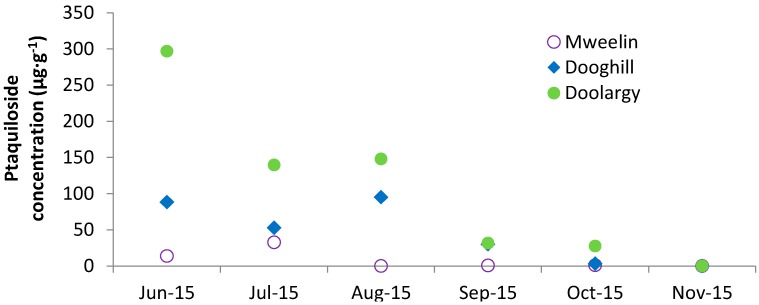
Trends in average ptaquiloside concentrations in bracken fronds from Mweelin Well, Dooghill and Doolargy.

A temporal pattern was observed in ptaquiloside concentrations from bracken fronds at the three drinking water abstraction sites (Figure 4). The highest frond concentrations were reported in June 2015. Concentrations began to decrease in September 2015 with a rapid reduction in ptaquiloside (<0.01 µg·g^−1^) in November 2015.

**Figure 5:**
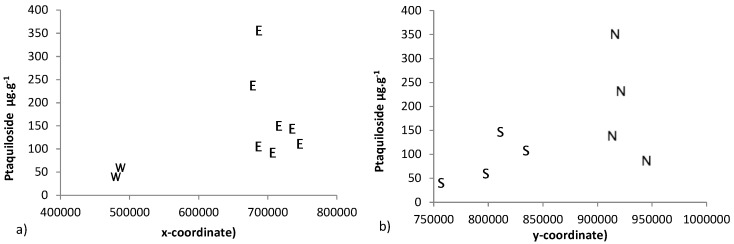
Scatterplots of ptaquiloside concentrations (µg·g^−1^) *versus* (**a**) x and (**b**) y coordinates (N—north; S—south; E—east; W—west) for fronds sampled in July 2015; Mweelin Well, Dooghill, Doolargy, Whitepark Bay, Glenarm, Churchtown, Drumsurn, Bann Estuary and Murlough.

Collated data on ptaquiloside concentrations in bracken fronds from the three abstraction sites and the six bracken management plots in Northern Ireland revealed spatial variation with lower concentrations of ptaquiloside in the west compared with the east of Ireland (Figure 5a). Similarly lower concentrations were observed in the south compared to the north of the country (Figure 5b).

### 2.3. Ptaquiloside in Surface Water and Groundwater Abstractions

Ptaquiloside was observed in samples from all three drinking water abstraction sites (Mweelin Well, Dooghill and Doolargy) in October 2015 (Table 2). The highest ptaquiloside concentration, 0.67 µg·L^−1^, was detected in Dooghill in the surface water stream, followed by 0.57 µg·L^−1^ in Mweelin and 0.01 µg·L^−1^ in Doolargy.

### 2.4. Bracken Management Plot Study

Multivariate statistical analysis on the bivariate response vector (density, height) showed an overall effect of site (*p* < 0.001), an effect of treatment (*p* < 0.002), an effect of time (*p* < 0.001) and of an interaction between time and treatment (*p* < 0.001). Regarding the univariate statistical tests of the effect of the input variables on each of the two responses separately, time effected both density and height (*p* < 0.001 in both cases) and there was evidence of a time*treatment interaction effect on each of these two responses (again *p* < 0.001 in both cases).

Average frond densities (number of fronds per m^2^ or # m^−2^) from all plots at all sites ranged between 51 fronds (July 2014—before treatment) and 1 frond per m^2^ (July 2015—after treatment). The maximum mean frond height recorded was 1160 mm in Churchtown in the control plot in 2015. The tallest fronds and highest densities were found in Murlough, Drumsurn and Churchtown, in 2014, prior to treatment.

**Figure 6:**
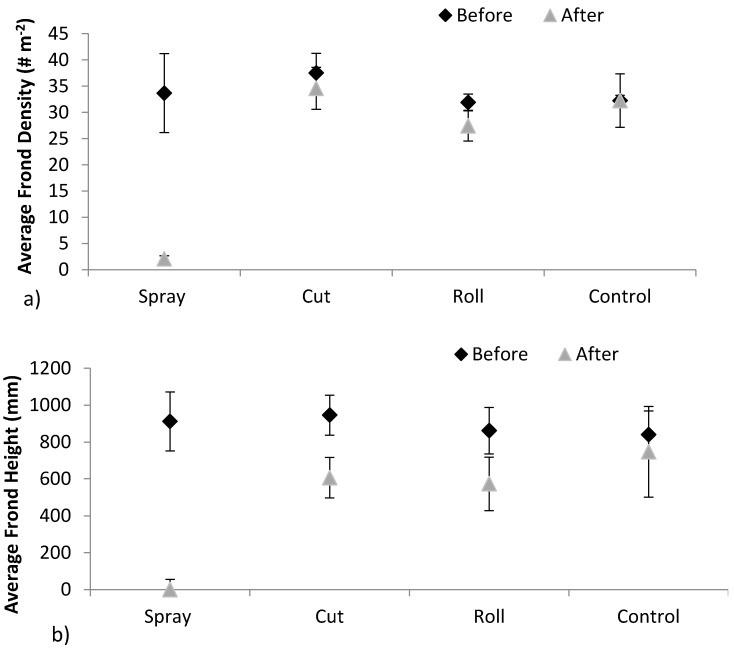
Bracken growth trends in (**a**) frond density and (**b**) frond height averaged across the 6 plot sites Whitepark Bay, Glenarm, Churchtown, Drumsurn, Bann Estuary and Murlough before and after treatment. (Error bars indicate standard deviations).

**Figure 7:**
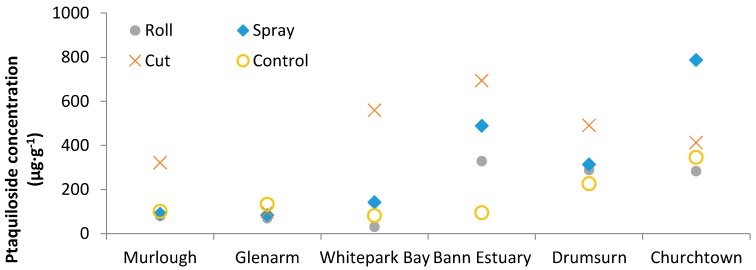
Ptaquiloside concentrations in bracken fronds at the 6 sites in Northern Ireland, UK, after treatment (roll, cut, spray).

Generally, frond densities were found to be consistent with earlier studies from the UK, but high relative to Danish studies (22). Frond lengths were shorter than observations from Britain and Denmark (22). Average frond densities differed significantly before and after treatment in the sprayed plots; however significant differences were not observed in the other treatment plots from before to after treatment (Figure 6) (*p* < 0.001). Average frond heights differed significantly in all plots except the control from before to after treatment, with the most significant difference observed in the sprayed plots (Figure 6) (*p* < 0.001). Ptaquiloside was detected in all frond samples analysed from the six treatment plots in Northern Ireland in July 2015 (Figure 7). Ptaquiloside concentrations ranged from 30.2–787 µg·g^−1^. The highest concentrations of ptaquiloside in fronds were observed in Churchtown and Bann Estuary (Figure 7).

Bann Estuary was most sensitive to the treatments possibly owing to the fact that it had previously being subjected to spraying. Overall, concentrations of ptaquiloside were markedly elevated in the plots subjected to cutting compared to the control plots with the notable exception of Glenarm where the different treatments had very little impact on ptaquiloside concentrations, and at Churchtown, where spraying had a much larger impact on the ptaquiloside concentrations in the fronds.

**Figure 8:**
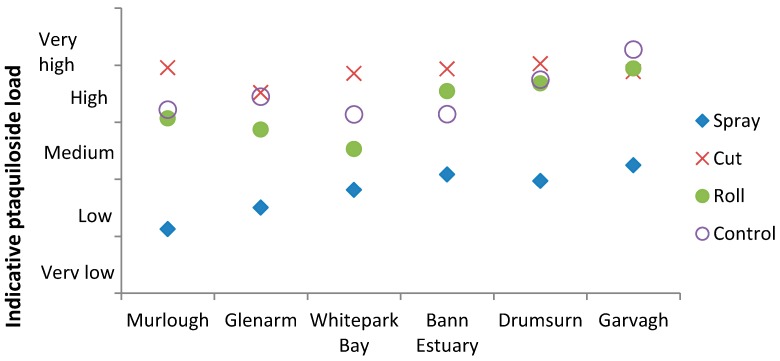
Effect of treatment regime on ptaquiloside load for the 6 experimental sites in Northern Ireland, UK.

The environmental load of ptaquiloside is a function of the frond concentration and the mass of bracken. In this study the height of the bracken was used as an indicative measure of its mass and an estimate of the ptaquiloside load was calculated as follows: load = function (height, density, ptaquiloside concentration). As the results are indicative of PTA load, they have been categorised from very low to very high rather than providing absolute values which could infer a greater accuracy than is real. Compared to the results given as concentrations (Figure 7), it is evident that, when the quantity of bracken is considered in conjunction with the ptaquiloside concentration, spraying has the largest significant effect on reducing ptaquiloside load (µg·m^−2^) (*p* = 0.005) and the impact of the cutting regime is less pronounced (Figure 8).

### 2.5. Discussion

Research underpinning quantification of ptaquiloside, and associated risk to humans and animals has expanded in the past decade, with most recent studies reporting on the fate of ptaquiloside in receiving water [19,33], animal plasma, urine and milk [34]. The proximity of bracken to drinking water supply sources and the absence of any relevant data on ptaquiloside in Ireland prompted the current investigation which has provided further evidence on the distribution of ptaquiloside in the environment.

In this study, above-ground fronds emerged in late-May as opposed to early-May as was observed in the UK [22], and dieback occurred in late-October to November. Frond emergence is expected to vary between years and sites [35] with varying temperature. Ptaquiloside was found in all frond samples analysed from June to October 2015 with concentrations ranging from 0.01–787 µg·g^−1^. These values are in line with previously reported values from other countries. A temporal trend in bracken frond ptaquiloside concentrations was observed with maximum frond concentrations occurring in summer and a sudden decrease observed in September. Spatial variation was also observed with lower concentrations of ptaquiloside observed in the west of Ireland compared with concentrations from the north and east of Ireland. This could be owing to regional climatic differences, the west of Ireland sites received greater annual average precipitation (1600–2000 mm·yr^−1^) than the north and east of the country (900–1500 mm·yr^−1^). Decreasing ptaquiloside concentrations in fronds are most likely due to rainfall wash-off and plant die off. The observed decrease of ptaquiloside in fronds in September coincided with peak annual rainfall. This trend is consistent with previous studies from Denmark and the UK [22,29] where ptaquiloside translocation to the roots and rhizomes has also been suggested [36]. Ptaquiloside concentration in bracken fronds has been shown to be a function of frond height and light-exposure [24]. However, no such relationship was found in this study and it is likely that several factors, including bracken management affect the typical pattern of ptaquiloside concentrations in the fronds.

Ptaquiloside presence was recently confirmed in Danish groundwater and surface water and the risk of human exposure through consumption of shallow, untreated groundwater was highlighted [19]. In our study, ptaquiloside was observed at all three drinking water abstraction sites in October 2015. This study is the first to show that ptaquiloside was present in surface water and groundwater drinking water abstraction sources located in bracken stands. The highest ptaquiloside concentration in bracken fronds surrounding the drinking water abstractions was observed at Doolargy (297 µg·g^−1^) in June 2015. In October 2015, the ptaquiloside concentration in bracken fronds at Doolargy (27.6 µg·g^−1^) was an order of magnitude higher than at Dooghill (3.33 µg·g^−1^), yet ptaquiloside concentrations in the receiving water were lower at Doolargy (0.01 µg·L^−1^) than at Dooghill (0.67 µg·L^−1^), which implies that the dynamics of ptaquiloside release, transport and degradation between the bracken source and water recipient, to a high extent, govern the observed concentrations. The measures of ptaquiloside in the bracken plant provide evidence that there is a significant source of ptaquiloside in the environment in Ireland, but the risk that this may pose to drinking water depends on other factors, such as soil type and proximity to the water abstraction point, and there can be significant decreases in the passing of ptaquiloside to soil layers with groundwater [19], which can be attributed to both microbial degradation and abiotic hydrolysis [37]. Dooghill was a surface water abstraction site whereas the other sites were spring wells and classed as groundwater. Water leaching to groundwater has a greater opportunity for degradation due to the time taken to infiltrate and this may partly explain the higher ptaquiloside concentrations in the surface water at Dooghill, but further studies would be required to verify this.

The pH of the soil underlying the bracken stands was slightly acid and hence is also in the range where ptaquiloside hydrolysis is minimal. Soils such as found at these study sites (peaty gleys) would further be expected to show less degradation as oxygen availability is reduced. Air temperature was ~3 °C lower in October than in September and low temperature contributes to the preservation of ptaquiloside. The appearance of ptaquiloside in October is most likely caused by autumnal rain flushing bracken fronds, a marked decrease in temperatures which reduces microbial activity in water aiding the preservation of ptaquiloside, and translocation of ptaquiloside to rhizomes coinciding with wilting of the plant [30]. Soils are wetter during this time and there is more rapid transport to and in surface water and groundwater. The groundwater sites in this study are “spring wells” and both sites are reported as having “extreme” groundwater vulnerability. Extreme groundwater vulnerability suggests these wells could be easily compromised by surface water contaminants.

25% of public drinking supplies in Ireland are derived from groundwater sources [38], with this figure rising to 100% in some regions. An estimate of approximately 200,000 private wells in Ireland [39] is supported by recent CSO data, which recorded 170,000 active domestic wells [40]. There is a lack of data on the exact locations of these private drinking water sources [41] which prevents a more accurate and large scale assessment of the risk posed by ptaquiloside. Private water supplies are likely to occur in areas not served by a mains supply. The three private water supplies examined in this study did not have any form of treatment and this is common of private water supplies in Ireland (where, when treatment is present, it is usually some form of water softener [42]). It is unlikely that water softeners will degrade ptaquiloside to any significant extent however further research would need to confirm this. There is a lack of measured data quantifying the efficacy of common treatment processes, for both private and public water supplies, with respect to ptaquiloside. Consequently, there is a need for increased public awareness relating to the potential occurrence of natural toxins in drinking water sources. Sampling ptaquiloside in water samples is still very much in its infancy and recent advances [33] in preserving samples taken in the field will assist in more realistic ptaquiloside observations.

Ptaquiloside concentrations are higher in young fronds compared to mature fronds [22,43,44] and it has been postulated that cutting bracken could increase the amount of ptaquiloside in the regrowth [23], a theory that has subsequently been verified [22]. However, this is the first study to assess the impact of mechanical and chemical bracken control methods on the phytotoxin content of bracken fronds. Spraying had the strongest effect on reducing the ptaquiloside load, and there was evidence that, at some sites, cutting increased the amount of ptaquiloside which is in accordance with the findings of Rasmussen *et al*. [22]. In the long term, bracken control should reduce the amount of bracken available and hence the risk that ptaquiloside may pose to drinking water. However, recent studies have demonstrated that it takes over 10 years to fully control bracken [45], and, with the recent ban on asulam, land managers are reverting to mechanical control techniques. Cutting and rolling are typically carried out one to three times a year for several years. Cutting may increase the environmental ptaquiloside load, although further studies are required to investigate the long term impact of cutting on bracken and ptaquiloside. Where mechanical means of control is not viable from an economic or safety perspective, bracken, and hence the potential risk from ptaquiloside will, at the least, remain, but it is more likely to expand due to the tenacious nature of bracken.

Knowledge of land management practices on the mass of bracken and its ptaquiloside content is required to make an informed assessment of the potential risks to drinking water. Increased awareness is imperative to land use managers on the implications of managing land with extensive bracken stands and also to owners of water supplies, both private and public, where water is collected in bracken-infested catchments, particularly where surface water abstraction occurs.

## 3. Experimental Section

### 3.1. Drinking Water Abstraction Sites

It was anticipated that we would follow the approach used by Ramwell *et al.* [23] to create a high risk catchment map for bracken in Ireland. However, owing to a lack of spatial data on national bracken percentage cover, information regarding bracken stands collated from the National Parks and Wildlife Service, following which drinking water abstraction information was obtained from local water managers. Three locations with established bracken stands were selected for the study (Mweelin Well, Dooghill, Doolargy, Figure 2). Mweelin Well and Doolargy sites had groundwater (spring well) abstractions and Dooghill had a surface water abstraction from a first order stream. Sampling was undertaken at all sites within two consecutive days in the last week of each month between June 2015 and November 2015, yielding a total of six water samples and six vegetation samples.

### 3.2. Bracken Management Plots

Six bracken stands located across Northern Ireland, UK, were selected for this investigation (Whitepark Bay, Glenarm, Churchtown, Drumsurn, Bann Estuary, Murlough, Figure 2; Table 1). At all sites, bracken coverage was dominant. Plots of approximately 20 × 40 m wide were established at each site. Four treatments were applied in July 2014. The treatment design was as follows: Control—no treatment; Roll; Cut; and Chemical—spraying with the chemical asulam. Asulox^®^ (a soluble concentrate containing 400 g·L^−1^ (33.6% *w*/*w*) of the sodium salt of asulam) was applied at a rate of 11 L·ha^−1^ in 400–500 L·ha^−1^ of water using an all-terrain vehicle (ATV) mounted boom sprayer. Bracken was cut using a flail cutter mounted on an ATV and rolled with a Cambridge type roller. Debris was not removed. Fronds were counted in eight 0.25 m^2^ quadrats in each plot before treatment (July 2014) and after treatment (July 2015) to estimate the frond density. The average height of the fronds in each treatment was also noted. Cutting and rolling were repeated in July 2015 following frond density measurements. Bracken was collected for ptaquiloside content on one occasion on the 23^rd^ of July 2015 at all six sites, two weeks after the repeat treatments (cutting and rolling) had been applied. Ten whole fronds were harvested for analysis at the soil surface from each treatment at each site. All 10 fronds were pooled to give a single, composite sample for each treatment at each site (*n* = 24).

### 3.3. Sampling and Extraction Protocol

The sampling and extraction protocols used the most recent developments from other researchers investigating ptaquiloside [33,46]. Water samples were collected in 100 mL amber bottles and buffered with 2.5 mL of 0.3 M ammonium acetate buffer, adjusted to pH 5 with glacial acetic acid [33]. Buffered samples were chilled on ice packs in the dark until further analyses were performed, with a maximum storage time of 1 day.

Vegetation samples comprised ten randomly harvested fronds, cut at the soil surface, from the dense bracken stand surrounding the drinking water abstraction. Each composite vegetation sample was placed in an opaque polyethylene bag and placed in a cooler box. The following day, pinnulae were stripped off and hard rachis were discarded [46]. Samples were mixed to homogenise and a subsample of fresh blades (6 g) was extracted twice with 90 °C MilliQ water (200 mL) in a fast rotating blender for 5 min. After filtration through cheesecloth (unbleached food grade) and immediate cooling of filtrates in an ice-water bath (5–8 °C), extracts were combined and centrifuged at 503 g for 5 min. After separation of solids, the volume was adjusted to 500 mL with 5 °C MilliQ water from which a 20 mL aliquot was drawn. Aliquots of the extract were analysed on the same day.

Three replicate 300 mm deep soil cores were collected from each of the study sites using a 30 mm diameter gouge auger. The vegetation layer was removed and the samples were homogenised. Three subsamples were obtained from each.

### 3.4. Chemical Analysis

Samples (water and vegetation extracts) were pre-concentrated by a factor of 20. A Solid Phase Extraction (SPE; 150 mg OASIS MAX Waters Corporation, (Milford, MA, USA) column was conditioned with 2 mL of methanol followed by 2 mL of MilliQ water (12). 20 mL of water/vegetation extracted sample was loaded on the column for cleaning and pre-concentration. The column was rinsed with 2 mL of MilliQ water followed by 2 mL of 15 % (*v*/*v*) methanol. Elution was performed using 2 × 0.25 mL of 80 % (*v*/*v*) methanol. Prior to analysis, the eluate was diluted 1:1 with MilliQ water to obtain a better performance on the LC column (12). External standard of ptaquiloside was isolated and purified from dried bracken material using the procedure described in Aranha *et al.* [34]. To evaluate the recovery of the method, 20 mL of MilliQ water was spiked with ptaquiloside at concentrations of 1 µg·L^−1^ for QC Low and 10 µg·L^−1^ for QC High.

A 6460 Triple Quad LC-MS (Agilent Technologies, Santa Clara, CA, USA) coupled with an Agilent 1200 HPLC system and interfaced with jetstream ionisation was used for the chromatographic separation and detection of ptaquiloside. Isocratic separation was performed using a 50 × 2.1 mm inner diameter, 1.8 µm, Zorbax SB-C18 porous shell rapid resolution high definition column (Agilent Technologies, Santa Clara, CA, USA) and eluent of 0.1% (*v*/*v*) formic acid. The flow rate was set to 0.3 mL·min^−1^. The retention time was 0.6 min. The column temperature was set to 45 °C. The samples were kept at 6 °C in the dark in the auto-sampler. Jetstream ionisation was used in negative mode. The capillary voltage was 3500 kV; the sheet gas flow (N_2_) was 11 L·min^−1^; and the carrier gas flow (N_2_) was 7 L·min^−1^, both with a pressure of 45 psi. The source temperature was 300 °C. Nitrogen was used as collision gas for MS/MS; collision energy and fragmenter voltage was optimized for the compound. Selected reaction monitoring (SRM) of *m/z* 421.1→241.1 were recorded and the collision energies was set to 5 eV. Fragmenter voltage was set to 100 V. The limit of detection for the overall method including SPE pre-concentration was 0.01 µg·L^−1^. The linear range of the LC-MS/ MS method was determined by triplicate injections of 7 standards in the range of 1–300 µg·L^−1^. Recovery for ptaquiloside was 70% for QC Low and 57% for QC High.

Soil samples were freeze dried at −50 °C (Freezone 12, Labconco, Kansas City, KS, USA) and pulverised in an agate mortar and pestle. Approximately 6 g of soil (dried, <2 mm sieved) was extracted with 30 mL of Morgans extractant (0.62 N NH_4_OH + 1.25 N CH_3_COOH), shaken for 1 h and filtered (Whatman^®^ Grade 1). Extracted P and K were measured by FIA (flow injection analysis) on a QuikChem^®^ 8500 Series 2 (FIA) Flow Injection Analysis System (Lachat Instruments, Milwaukee, WI, USA) and extracted magnesium was determined by atomic absorption spectrophotometry (spectrAA 200, Varian, CA, USA). Soil pH was measured in 1:5 soil–water suspensions.

### 3.5. Statistical Analysis

Differences in frond density (# m^−2^) and frond height (mm) were tested with a before-after-control-impact design. The response of ptaquiloside in the bracken fronds were examined across the different treatments and six sites. An indicative estimate of the indicative ptaquiloside load was calculated as the product of ptaquiloside concentration (µg·g^−1^) by calculated biomass (g) by density (# m^−2^), with height and density being a substitute for total mass of bracken per metre square. In order to calculate the absolute load, a mass of bracken is required, but these data were not available. Other researchers have generated transfer functions to estimate bracken biomass from frond length [22,36] but it is not known whether these functions would be valid for the current study, particularly as there were different functions for managed *cf* unmanaged bracken. In this study, it could be argued that the control bracken was unmanaged whilst the other plots managed, yet it would not be correct to apply different transfer functions under a single test system. Using transfer functions created by others would introduce an unknown error, and the presentation of the load in µg.m^−2^ using such functions would infer an accuracy that was not present. For these reasons, the ptaquiloside load has been estimated to provide an indicative value using the measured data available (ptaquiloside concentration, frond height and frond density). This approach is valid as the data are used for comparative purposes.

A General Linear Model (GLM) with site (Whitepark Bay, Glenarm, Churchtown, Drumsurn, Bann Estuary and Murlough) and treatment (control, roll, cut and spray) as between subject variables (and possible interaction effects among these) was fitted to study the effects on the response ptaquiloside load. Additionally, a GLM with time (before and after treatment) as a within subject variable and site (Whitepark Bay, Glenarm, Churchtown, Drumsurn, Bann Estuary and Murlough) and treatment (control, roll, cut and spray) as between subject variables (and possible interaction effects among these) was fitted to study the effects on the responses density (# m^−2^) and height (mm).

Correlation which would exist between observations taken before and after was accommodated by using a repeated measures analysis for this factor. In addition, an improvement on the standard analysis was achieved as follows: While it would be possible to analyse density and height as separate responses using the above within and between factors, a more powerful analysis can be achieved by capitalising on the correlation that should exist between these two responses. Thus, a multivariate analysis was performed which not only studies the effect of the input variables on the joint response but also permits univariate results on each of the two responses separately. As indicated above, in the spirit of multivariate statistical analysis, these two univariate analyses tend to give more powerful results than analysis of each variable separately would have allowed.

Residual diagnostics were conducted to examine any violations of assumptions underlying the linear model that are needed to justify the analysis. To accommodate possible violation of assumptions, we could have transformed the data but prefer to report our results as somewhat exploratory , especially in light of the relatively small sample sizes at each combination of values of the input variables. Statistical analyses were conducted with IBM SPSS 22.0 [47] and Minitab 17 Statistical Software [48].

## 4. Conclusions

The importance of natural toxins such as ptaquiloside from bracken has been recognised in recent years. Coinciding with this has been the recent ban on one of the most effective herbicides to treat bracken ferns, and there is an expectation of widespread bracken invasion. This study has shown that ptaquiloside is present in bracken fronds in Ireland from frond emergence (14.1–297 µg·g^−1^) to plant die off (0.03– less than 0.01 µg·g^−1^), and can be found in concentrations as great as 0.67 µg·L^−1^ in drinking water from abstractions sites. There is a suggestion that spatial and temporal variation exists; however, further research with an increased number of sites and a higher temporal resolution is warranted to confirm this. Spraying was the most effective means of control for reducing ptaquiloside load whereas cutting bracken may increase the production of ptaquiloside in the short term and hence the potential risk to drinking water. Education of owners of private water supplies and land managers, as to the risks of natural toxins is required to minimise health risks.

## Figures and Tables

**Figure 3:**
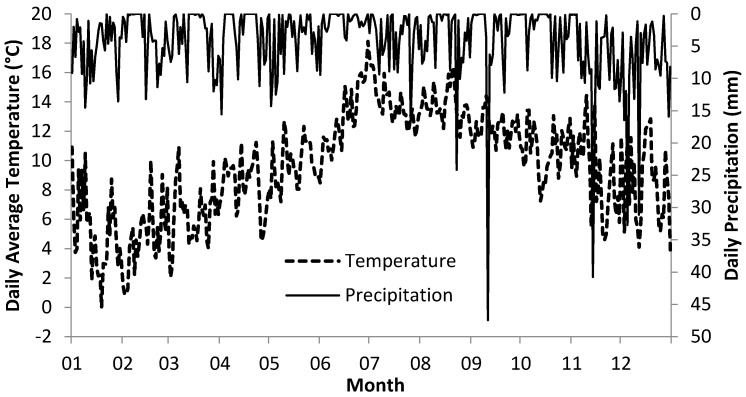
Daily average temperatures and daily total precipitation (mm) from January to December, 2015. These readings are taken from averages of two Met Eireann synoptic weather stations (See Figure 2).

**Table 1:** Study site locations and characterisations.

Site Name	x-Coordinate	y-Coordinate	Altitude (m.a.s.l.)	Soil Type	Landuse	Soil Available P (mg·kg^−1^·dw)	Soil Available K (mg·kg^−1^·dw)	Soil Available Mg (mg·kg^−1^·dw)	Groundwater Vulnerability	Average Annual Precipitation (mm)	Soil pH
Murlough ^A^	740704	835420	15	Poorly drained gleys	Nature Reserve	n/a	n/a	n/a	n/a	900–1000	5.30
Glenarm ^A^	729329	913040	52	Poorly drained gleys	Sheep grazing	n/a	n/a	n/a	n/a	1250–1500	6.00
Whitepark ^A^	701135	944416	14	Poorly drained gleys	Cattle grazing	n/a	n/a	n/a	n/a	1000–1250	7.50
Drumsurn ^A^	672517	920998	86	Poorly drained gleys	Cattle grazing	n/a	n/a	n/a	n/a	1000–1250	5.40
Bann Estuary ^A^	680615	935318	6	Poorly drained gleys	Cattle grazing	n/a	n/a	n/a	n/a	1000–1250	5.50
Churchtown ^A^	681061	915631	84	Poorly drained gleys	Cattle grazing	n/a	n/a	n/a	n/a	1000–1250	5.30
Dooghill ^B^	482372	798514	25	Peaty podzols	Sheep grazing	2	35	78	Extreme	1600–2000	5.64
Doolargy ^B^	710012	811895	148	Peaty podzols	Sheep grazing	3	96	109	Extreme	1000–1200	5.82
Mweelin ^B^	475591	757708	68	Peaty podzols	Sheep grazing	<1	63	176	Extreme	1600–2000	6.86

^A^ denotes bracken management plots in Northern Ireland, UK; ^B^ denotes the water abstraction sites, Ireland; soil available P and K were determined by Flow Injection Analysis System and Mg was determined by atomic absorption spectrophotometry for the upper 300 mm soil layer. Daily average temperatures ranged between −0.01 and 18.1 °C in 2015 with maximum temperatures observed in July and minimum temperatures observed in January (Figure 3). Daily precipitation ranged between 0 and 47.5 mm with highest rainfall observed in September 2015.

**Table 2:** Ptaquiloside concentrations (µg·g^−1^) in bracken fronds and water samples from the drinking water abstraction sites; and total rainfall (mm) and average temperature (°C) in the twenty-four hours preceding sample collection taken from Met Eireann rainfall stations adjacent to each of the study sites.

Site	Jun-15	Jul-15	Aug-15	Sep-15	Oct-15	Nov-15
**Vegetation ptaquiloside (µg·g^−1^)**						
Mweelin Well	14.1	33.1	0.27	0.88	1.34	<0.01
Dooghill	88.4	52.7	95.1	30.0	3.33	0.03
Doolargy	297	140	148	32.0	27.6	0.01
**Water ptaquiloside (µg·L^−1^)**						
Mweelin Well	0.00	0.00	0.00	0.00	0.57	0.00
Dooghill	0.00	0.00	0.00	1.25	0.67	0.00
Doolargy	0.00	0.00	0.00	0.00	0.01	0.00
**Rain (mm)**						
Mweelin Well	3.60	7.00	12.40	0.00	6.80	3.40
Dooghill	0.00	1.20	2.60	0.00	3.80	2.20
Doolargy	0.20	10.60	0.10	0.00	11.10	0.10
**Temperature (°C)**						
Mweelin Well	16.53	14.23	14.43	16.23	13.00	9.85
Dooghill	17.18	13.75	13.63	18.15	12.20	9.85
Doolargy	23.70	15.30	13.53	16.13	12.75	9.85
